# The dawn has come for new therapeutics to treat atherosclerosis: Targeting neuroimmune cardiovascular interfaces in artery brain circuits

**DOI:** 10.1002/ctm2.1040

**Published:** 2022-09-02

**Authors:** Sarajo Kumar Mohanta, Christian Weber, Changjun Yin, Andreas J. R. Habenicht

**Affiliations:** ^1^ Institute of Cardiovascular Prevention Ludwig‐Maximilians‐University (LMU) Munich Munich Germany; ^2^ DZHK (German Centre for Cardiovascular Research) Partner Site Munich Heart Alliance Munich Germany; ^3^ Munich Cluster for Systems Neurology (SyNergy) Munich Germany; ^4^ Department of Biochemistry Cardiovascular Research Institute Maastricht (CARIM) Maastricht University The Netherlands; ^5^ Institute of Precision Medicine The First Affiliated Hospital of Sun‐Yat‐sen University Guangzhou Guangdong China

Atherosclerosis is the leading cause of mortality worldwide. However, regrettably, there are no treatment options that target the root causes of the disease, as its pathogenic mechanisms largely remain to be defined. Atherosclerosis has been viewed as a chronic inflammatory and unresolvable condition of all three arterial wall layers: Plaques develop in the inner intima layer of arteries leading to lumen narrowing and reduction of blood flow to downstream tissues, and when critical thresholds are breached, they cause heart attacks and strokes; smooth muscle cells in the media layer transdifferentiate into a synthetic and migratory phenotype and participate in artery remodelling by changing their transcriptome profile and secreting cytokines; and immune cells accumulate in the outer connective tissue coat of arteries, that is, the adventitia, and adopt roles in atherosclerosis‐specific immune responses. Since atherosclerotic plaques are not innervated, until recently, the hardwired connections between the peripheral nervous system (PNS), the central NS (CNS) and the arterial wall remained unknown. However, since the NS uses the adventitia of all arteries as its main conduit to reach distant targets and indeed all parenchymal cells, we speculated that PNS axons may crosstalk to adventitia immune cells. Unexpectedly, we found that widespread neuroimmune cardiovascular interfaces (NICIs) arise in atherosclerosis‐diseased adventitia segments and that both the sensory NS and the sympathetic NS (SYNS) establish a structural artery–brain circuit (ABC): its sensory arm enters the CNS via dorsal root ganglia, and from there, polysynaptic artery‐brain projections connect to higher brain regions including the parabrachial nucleus and the central amygdala; and the efferent arm of the ABC project from hypothalamic neurons back to the adventitia via bifurcated projections involving the parasympathetic NS and the SYNS. Moreover, celiac ganglionectomy reduces disease progression and stabilises plaque vulnerability. Thus, the PNS uses NICIs to assemble a structural ABC, and therapeutic intervention into the ABC attenuates atherosclerosis. Here, we outline how this previously unknown hardwired connection of arteries with the CNS may lead to new classes of therapeutics.

## 1 NICIs ESTABLISH A NEW ATHEROSCLEROSIS DISEASE PARADIGM

**FIGURE 1:**
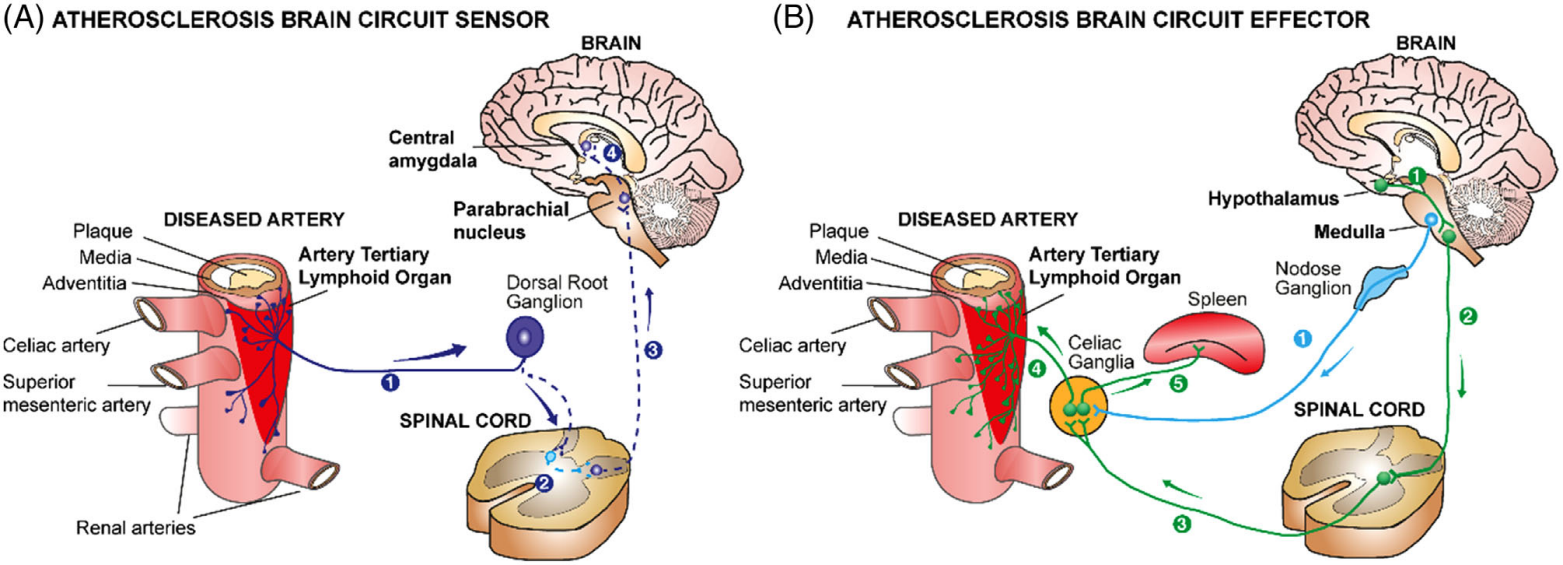
Atherosclerotic arteries are connected to the SENS and the SYNS to form an ABC. (A) Adventitia NICIs initiate the ABC using sensory neurons of dorsal root ganglia (DRGs) (❶), then connect via the contralateral dorsal horn of the spinal cord via interneurons (❷) to reach the parabrachial of the medulla (❸) and the central amygdala (❹). (B) SYNS efferents of the ABC effector project from hypothalamic and brainstem nuclei (❶) to the spinal cord (❷) and from there project to the ipsilateral ventral horn to PNS ganglia including the celiac ganglion (❸) to synapse to SYNS neurons to directly innervate the adventitia (❹). Within the celiac ganglion, the splenic nerve projects to the spleen to affect splenic immune responses (❺). For ease of reading, we did not depict multiple efferent projections from the celiac ganglionic plexus to multiple organs including the gastrointestinal tract. In addition, vagal efferents originating in the medulla oblongata project to the celiac ganglion, after traversing the nodose ganglion in the neck to innervate multiple internal organs (❶)

**FIGURE 2:**
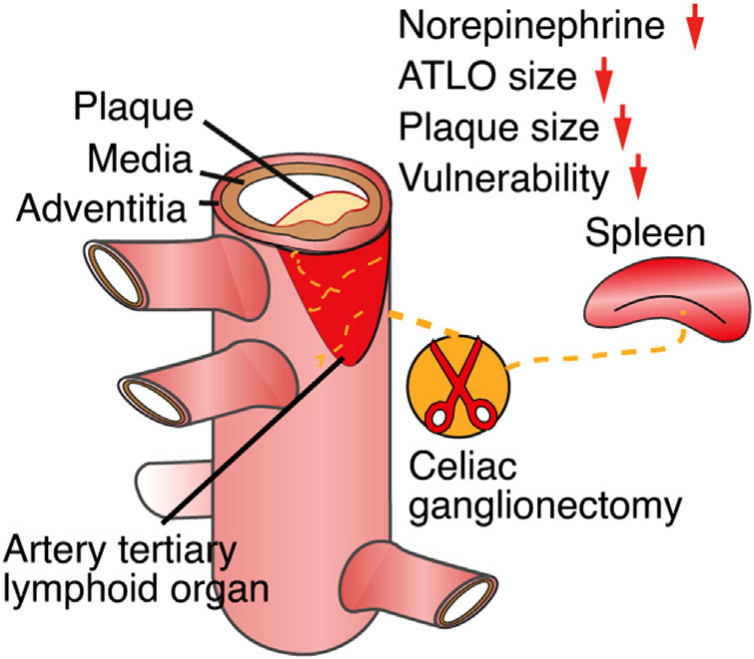
Celiac ganglionectomy attenuates atherosclerosis progression. Surgical removal of the celiac ganglion attenuates atherosclerosis progression, leads to ATLO disintegration and diminishes adventitia norepinephrine levels

Our recent data in experimental mice and human cardiovascular tissues^1^, ^2^ have provided information on a series of unexpected mechanistic insights into the pathogenesis of atherosclerosis.^3^, ^4^, ^5^ This body of evidence suggests a new disease paradigm of atherosclerosis progression. We propose that adventitia NICIs are proxy sentinel sensors and effectors of atherosclerosis created by long‐lasting interactions of the PNS with both the immune and vascular systems in tripartite tissue interactions.^1^ Using virus tracing techniques, we reconstructed a disease‐specific polysynaptic hard‐wired PNS‐CNS connectivity network to form a structural ABC (Figure 1). The initiating event to establish this ABC appears to originate in plaques throughout the arterial tree: here, aggregates of immune cells accumulate in adventitia segments adjacent to plaques but not in disease‐free segments. The adventitia NICI and its connection to the brain therefore appear to be a systemic phenomenon encompassing the entire arterial tree. Over time, a multisynaptic ABC emerges during adulthood and ageing, including a sensory NS (SENS) arm, and bifurcated SYNS and parasympathetic NS (PANS) effector arms (Figure 1). Moreover, therapeutic intervention into the SYNS attenuates atherosclerosis (Figure 2). Although neuroimmune interactions^6^ have been described previously including those in cancer,^7^ obesity,^8^ thermoregulation,^9^ brain diseases^10^ and inflammatory bowel diseases^11^, ^12^ the identification of the ABC may establish a new disease paradigm for atherosclerosis. It addresses key features of neuroimmunology^13^, ^14^, ^15^ in atherosclerosis pathogenesis and integrates the vascular system as a core third systemic participant.^16^ Indeed, the vascular system qualifies for a dual role in tripartite rather than bidirectional tissue interactions in atherosclerosis: adventitia segments adjacent to plaques speak with the SENS and the SYNS by providing a biological platform to sense plaque‐derived molecular information via neuroimmune junction formation to transient receptor potential (TRP)‐ type nociceptors capable of translating inflammatory cytokine‐derived signals into action potentials at axon endings which are ultimately projected to the brain.^6^, ^17^ On the intima side of the arterial wall, lumenal endothelial cells receive signals from the circulation resulting in plaque growth and, eventually, trigger clinically significant diseases such as heart attacks and strokes.^3^, ^16^ As none of the multiple interactions between the vascular system, the immune system and the NS have been known until recently, the possibility of new therapeutic strategies deserves careful consideration.

## 2 NEW THERAPEUTIC TARGETS WITHIN THE ABC SENSOR

Adventitia NICIs so far studied are characterised by marked axon neogenesis and restructuring involving axons of both the SENS and the SYNS but we did not observe axons in the adventitia PANS until now. At a cellular level, multiple immune cells in the adventitia have been identified: they include myeloid cells including macrophage subtypes, dendritic cell subtypes, and both types of adaptive immune cells and their subtypes, that is, B cells and T cells. Adventitia NICIs markedly alter signalling molecules of cells involved in the interaction of a large number of participating cells with axon tips of the SYNS and the SENS as indicated by whole genome transcriptome and single cell RNA sequencing (scRNA‐seq) profiling.^1^ Using whole genome sequencing profiling of ganglia of the PNS has yielded a large number of potential targets for future therapeutic approaches including molecules and transcripts released at axon endings such as epinephrine, cytokines, synaptic proteins, regulators of axon neogenesis and inflammatory mediators.^18^ Many of these newly identified molecules are apparent target candidates for future treatments of late stage atherosclerosis. Yet, defining atherosclerosis‐associated molecules and studies of their impact on atherosclerosis progression is a daunting task because of their sheer number. Such studies will involve extensive further usage of preclinical mouse models as well as translational studies in human patients. Translational studies will need to focus not only on leukocytes in the adventitia adjacent to atherosclerotic plaques but they should seek to examine paraarterial ganglia, the nodose ganglia (a major SENS ganglion of the PNS) and DRGs to identify neuronal genes that may be altered during human atherosclerosis progression (Figure 1). Moreover, delineation of the cell‐cell crosstalk within NICIs has uncovered previously less known mediators of atherosclerosis progression at axon endings including axon‐derived neurotransmitters of the SENS such as calcitonin gene‐related peptide and the SYNS such as high tissue concentrations of norepinephrine but also those of Schwann cells such as nerve growth factor and many more. Many of these molecules have previously not been tied to atherosclerosis progression including regulators of axon homeostasis and inflammatory cytokines and their receptors. For example, we observed marked increases in TRPV1 expression at axon endings of SENS axons that are in close proximity to various types of adventitia immune cells most likely forming neuroimmune junctions. Axon tips expressing TRPV1 and possibly other sensory potential channels now need to be studied at functional levels. These include potential channels mediating signals of stretch, pain, heat, cold and inflammation. Our observation of apparent TRPV1 smooth muscle cell junctions indicates that the SENS may sense the inflammatory environment and transmit signals via the pain pathway in the brain raising the important possibility that TRP family members may be involved in atherosclerosis progression.^13^, ^17^, ^19^, ^20^, ^21^, ^22^, ^23^, ^24^, ^25^ We noticed earlier that there is also aberrant lymph vessel neogenesis, angiogenesis and de novo synthesis of high endothelial venules in ATLOs.^26^ It will be of interest to examine whether the altered lymph vessels in the adventitia adjacent to atherosclerotic plaques interact with components of the PNS and whether TRP channels are expressed in lymph vessels and newly formed blood vessels as well as in high endothelial venules. All these cells, molecules and structural components may turn out to be candidates for future therapeutic approaches. It is apparent, however, that a more detailed molecular and cellular characterization of NICIs—not to speak about the epigenetic landscape—will require major efforts in the years to come.

## 3 NEW THERAPEUTIC TARGETS WITHIN THE ABC EFFECTOR

As surgical therapeutic intervention into the SYNS of aged hyperlipidemic mice was shown to attenuate atherosclerosis progression and stabilise plaque erosion,^1^ we obtained proof‐of‐concept evidence that interference into components of the PNS can impact atherosclerosis progression (Figure 2). These observations call for a comprehensive delineation of other components of the PNS as well as the CNS that may affect disease progression. While such a proof‐of‐concept is conceptually important, there are caveats when considering the development of future therapeutic strategies. Some of these caveats need to be taken into consideration early to avoid flawed approaches going forward: that is, removal of the celiac ganglion and its relation to the function of adventitia axon networks is difficult to interpret in the sense that its spatial microanatomy is complex: removal of the celiac ganglion not only removes all axons within the adventitia but also takes out the splenic nerve which originates in the celiac ganglion and in addition it removes the innervation of the gastrointestinal tract. Thus, it has been shown that the spleen is an important regulator within the immune system to affect healing of the myocardium after experimentally introducing a myocardial infarct.^27^, ^28^ All the experimental evidence so far obtained regarding therapies in future human trials is by far not sufficient to justify the initiation of clinical trials any time soon. Much more information on mice has to be obtained to even outline potential strategies. From future preclinical studies in mice, much more has to be done to translate the most significant studies to humans. This translational work will require a large number of patients to stratify any concept to the more complex risk factor profile of humans with atherosclerosis versus atherosclerosis mouse models. However, human studies should be initiated soon and should involve the design of separate clinical cohorts relative to gender, age and risk factor profiles.

## 4 TAKING ADVANTAGE OF THE NICI‐TRIGGERED ABC TO DEVELOP NEW CLASSES OF THERAPEUTICS TO TREAT ATHEROSCLEROSIS

While it is too early to name specific targets and to examine their mechanisms of action, we can envision a few principles of future therapeutics:

**The innate immune system**: When considering interference into the innate immune system, therapies cannot conceivably come from a general understanding of atherosclerosis as an inflammatory disease per se or from some general view of the role of oxidation reactions including oxidised LDL for the pathogenesis of atherosclerosis. Any anti‐inflammatory disease interference strategy should be developed from and focused on a comprehensive understanding of the nature of inflammation in atherosclerosis, their participating cells and the specific pathways involved. Such a disease‐specific innate immune system response, however, may not exist including the IL‐1‐IL‐6‐CRP axis (see below the discussion on the CANTOS trial). In addition to the focus on the arterial wall, therapeutic approaches should possibly be tissue‐specific for example by targeting the liver as shown by the use of a liver‐specific small interference RNA oligonucleotide treatment strategy against the key inflammation‐related complement component C5 reported by our group.^29^ However, we are cautiously warning that interference into the innate immune system will presumably never be effective as molecular mechanisms and the contribution of distinct innate immune cell subtypes in atherosclerosis are hard calls to make. Moreover, anti‐inflammatory therapies are expected to be risk‐prone by predictable (wound healing delayed or immunosuppressive) but also may involve unpredictable side effects such as severe risks for virus infections.^29^ Therefore, atherosclerosis trials have to be conducted not only in patients burdened by atherosclerosis but also, in parallel, in patients afflicted with other clinically important conditions, that is, cancer, immunocompromised individuals (i.e. AIDS) and patients with autoimmune diseases.^30^, ^31^, ^32^ A large number of clinical trials targeting oxidation reactions including antioxidant vitamins such as vitamin C, D and E and other pharmaceuticals to target presumed proinflammatory pathways in atherosclerosis have failed indicating that targeting oxidation reactions may be flawed approach.^31^, ^32^ The Canakinumab Anti‐inflammatory Thrombosis Outcome (CANTOS) trial has recently received widespread attention triggering considerable enthusiasm among some but also received cautious comments among other members of the scientific community that anti‐inflammatory therapies targeting innate immune responses may turn out to be effective strategies for both primary and secondary prevention in patients afflicted with advanced stages of atherosclerosis.^31^, ^32^ We do not share this enthusiasm as the CANTOS trial did not achieve an important endpoint, that is, reduction of overall mortality and instead, as expected, it was associated with a significant increase in fatal infections.^31^, ^32^ Multiple other trials turned out to be disappointing painting a gloomy future for broad anti‐inflammatory therapies targeting the innate immune system to treat atherosclerosis.^31^, ^32^ Therefore, much more sophisticated and tailored approaches rather than broad approaches will be needed for future therapies.
**The adaptive immune system of B and T cells**: It will be challenging to select therapeutic targets and define risk factor‐prone patient cohorts related to adaptive rather than innate immune responses. However, these approaches hold major conceptually well explainable reasonings. First of all, the targets should be closely disease‐related, and their pathways to promote atherosclerosis well understood at a functional level in preclinical experimental models. Yet, similar to the approaches in innate immune responses, promising intervention to treat atherosclerosis by attempts to target a complete and broad particular T cell or B cell subtype appears to be flawed: the dichotomic and plastic nature of adaptive immune responses would imply that the immune system reacts to counterbalance these interventions. For example, T regulatory (T_reg_) cells can switch sides from an immunosuppressive to a proinflammatory phenotype to produce IL‐17 by a phenomenon termed immunoconversion^33^, ^34^ and similar off target effects are likely to occur in antigen‐unspecific B cell‐ or T cell‐directed therapeutics. What then can be done to target the adaptive immune system? Major promises regarding effective therapeutic approaches may evolve if atherosclerosis‐specific autoantigens can be identified particularly for T_reg_ cell‐directed therapies. Thus, it will be instrumental to identify potential arterial wall‐derived autoantigens and their antigen‐specific B cell receptors and/or antigen‐specific T cell receptors. Once such autoantigens will have been identified, the use of chimeric antigen receptor (CAR) gene editing approaches including the CRISPR‐CAS9 approaches become feasible to be tested in preclinical models of atherosclerosis and other unresolvable chronic inflammatory and autoimmune diseases.^35^ These approaches could lead to vaccination strategies, antibody (B cell‐based) treatment strategies and T cell therapies by engineering T cell receptors that specifically recognise disease‐causing peptide sequences in bona fide atherosclerosis‐specific autoantigens with the help of CRISPR‐CAS9‐gene engineered cells.
**The NICI‐triggered ABC**: For the development of new classes of treatments exploiting the NICI‐triggered ABC, it is too early to outline them in detail but the direction of such strategies become visible: Such approaches should identify molecules that are key promoters of the disease as derived from single cell sequencing approaches of tissues directly participating in the ABC, that is, the immune system, the NS and the cardiovascular system. As NICIs form in tissues where all three systems interact, it will be important to apply scRNA‐seq approaches to the known NICIs. Until today, we have observed three distinct NICIs in the PNS: the adventitia NICI, the SYNS ganglia NICI and the SENS NICI in DRGs.^1^ In addition to scRNA‐seq approaches that will yield important information on the modification of transcriptomes in all cells of PNS ganglia, we predict that NICIs exist in the CNS (Figures 1 and 2). Appropriate mouse models will have to be investigated combined with translational work to confirm key data in human cardiovascular tissues. To identify new therapeutic targets, the extensive use of Designer Receptors Exclusively Activated by Designer Drugs (DREADDs) will be needed. The DREADD approach is particularly suited to be applied in atherosclerosis as a slowly developing artery disease requiring months to develop as DREADs can act in a tissue‐cell type‐specific way for long periods of time. Although DREADDs are so far used in preclinical models as experimental tools there is work underway to develop the first DREADDs for human diseases.^36^, ^37^, ^38^

## 5 WHERE TO GO FROM HERE: TASKS AHEAD

Provide a connectivity map of the atherosclerotic PNS and the CNS using virus tracing methodologies.^39^

Identify atherosclerosis‐associated transcriptome modifications of PNS and CNS neuronal genes of crucial ABC territories in the atherosclerotic CNS in mouse models and human brains.

Test activation and inactivation strategies using DREADDs in mouse models of atherosclerosis

Use tissue clearing approaches to define the spatial relation of the immune system, the NS and the cardiovascular system

## 6 CONCLUDING REMARK

The discovery of a NICI‐triggered ABC opens the field of neurobiology to study atherosclerosis progression, define the underlying molecular mechanisms in the PNS and CNS and design and develop new classes of therapeutics in the hope to treat the root causes of clinically significant disease as well as develop therapeutics in primary prevention approaches.

## CONFLICT OF INTEREST

There is no conflict of interest.
